# Poor Law Topics

**Published:** 1906-03-10

**Authors:** 


### POOR LAW TOPICS.

### SUBSIDISING THE SWEATER.

A case which recently came before the Lambeth Guardians shows clearly that risk of lowering wages by giving out-relief which is one of the troubles of the Poor-law administrator. A woman who was applying for out-relief stated that, making Volunteer trousers for a firm of contractors, she could earn 11s. 8d. by 141 hours' work. It took her three hours' work to "finish" a pair of trousers, for which, when done, she received 2¾d.—less than a penny an hour. For years this woman had got up at three o'clock in the morning, worked at home until eight o'clock, then gone to a factory and worked there until night. Even then her average wages had been only 8s. to 9s. a week. Strength failing her to earn this, she asked for out-relief to make up, with her work, a pittance on which she could live. When anyone makes application for out-relief, the Guardians ask if they are in possession of sufficient income to cover their rent. Without that there is no alternative but the workhouse; but if that be met, either by the applicant's own work or by the charity of others, out-relief will be given, and very rarely is it asked how much work goes to earn the necessary few shillings. The Guardian who would not consent to this is said to be both hard-hearted—driving the respectable poor from their homes into an institution—and foolish; for the workhouse inmate costs far more per week than the recipient of out-relief. The poor creatures themselves cling to their attic or cellar, the little corner of the world which is all their own. They struggle on to get through the work, whose payment will qualify them for out-relief. Unscrupulous employers, knowing that their workers can count on this dole, bring down the wage to a point which will not permit the worker to live without it. The out-relief becomes practically a grant in aid of wages, and the worker who has not that outside aid can hardly, with the longest and severest labour, make enough to live on. The competition of the parish-aided worker is the worst handicap on free labour. The easy-going and short-sighted socialism which is at present popular among the unpaid administrators of Poor-law relief blind them to the underlying economic consequences of their actions. They fail to see that they are subsidising the sweater, and making that "living wage" of which they talk so much unattainable to non-paupers.

